# Ginsenoside Rb1 Blocks Ritonavir-Induced Oxidative Stress and eNOS Downregulation through Activation of Estrogen Receptor-Beta and Upregulation of SOD in Human Endothelial Cells

**DOI:** 10.3390/ijms20020294

**Published:** 2019-01-12

**Authors:** Jian-Ming Lü, Jun Jiang, Md Saha Jamaluddin, Zhengdong Liang, Qizhi Yao, Changyi Chen

**Affiliations:** 1 Michael E. DeBakey Department of Surgery, Baylor College of Medicine, Houston, TX 77030, USA; jian-ming.lu@bcm.edu (J.-M.L.); Jiangjun_64@163.com (J.J.); mdsjam@gmail.com (M.S.J.); zhendonl@bcm.edu (Z.L.); qizhiyao@bcm.edu (Q.Y.); 2 Center for Translational Research on Inflammatory Diseases (CTRID), Michael E. DeBakey Veterans Affairs (VA) Medical Center, Houston, TX 77030, USA

**Keywords:** ginsenoside Rb1, ritonavir, oxidative stress, endothelial nitric oxidase synthase, estrogen receptor, superoxide dismutase, endothelial dysfunction

## Abstract

We have previously shown that ritonavir (RTV), a highly active anti-retroviral therapy (HAART) drug, can cause endothelial dysfunction through oxidative stress. Several antioxidants including ginsenoside Rb1, a compound with antioxidant effect, can effectively block this side effect of RTV in endothelial cells. In the current study, we explored a mechanism by which ginsenoside Rb1 could protect these cells via binding of estrogen receptors (ERs). We found that several human endothelial cell lines differentially expressed ER-β and had very low levels of ER-α. RTV treatment significantly increased the production of reactive oxygen species (ROS) and decreased the expression of endothelial nitric oxidase synthase (eNOS) and superoxide dismutase (SOD) in HUVECs, while Rb1 effectively blocked these effects of RTV. These effects of Rb1 were effectively inhibited by silencing ER-β, indicating that ginsenoside Rb1 requires ER-β for its antioxidant activity in inhibiting the deleterious effect of RTV in human endothelial cells. Furthermore, Rb1 specifically activated ER-β transactivation activity by ER-β luciferase reporter assay. Rb1 competitively bound to ER-β, which was determined by the high sensitive fluorescent polarization assay.

## 1. Introduction

Recently, HIV infection and treatment with highly active antiretroviral therapy (HAART) have been associated with a high incidence of endothelial dysfunction and cardiovascular disease [1,2,3,4]. Approximately 36.9 million people worldwide are infected with human HIV-1, according to a World Health Organization report published in 2017, and 21.7 million people living with HIV were accessing HAART [5]. Although HAART has markedly reduced the morbidity and mortality associated with HIV-1 infection, cardiovascular complications have emerged as a great risk of death in HIV-1 infected patients [1,2,3,4,6]. Long-term HAART may cause oxidative stress and vascular dysfunction [6,7,8]. However, the exact molecular mechanisms of these complications and effective therapeutics are not fully understood. There has been a long-term interest in this field to determine the effects of antiretroviral drugs in the vascular system [9,10,11,12].

Endothelial dysfunction is the initial step in the pathogenesis of atherosclerosis and is characterized by decreased bioavailability of nitric oxide (NO), which may be due to enhanced NO catabolism secondary to increased superoxide anion (O_2_^●−^) production or reduced expression and/or activity of endothelial nitric oxide synthase (eNOS) [13,14,15]. One of the major underlying mechanisms of endothelial dysfunction is oxidative stress, whereby reactive oxygen species (ROS) are increased and NO bioavailability is reduced. ROS are a family of molecules produced via aerobic cellular respiration. ROS includes molecular oxygen and its derivatives, such as superoxide anion (O_2_^●−^), hydroxyl radical (•OH), nitric oxide (NO•), and lipid radicals [16]. Dismutation of O_2_^●−^ by endogenous antioxidant superoxide dismutase (SOD) produces the more stable ROS, hydrogen peroxide (H_2_O_2_), which is then converted enzymatically into H_2_O by catalase and glutathione peroxidase (GPX). H_2_O_2_ can be also scavenged by peroxiredoxins [17]. H_2_O_2_ can react with reduced transition metals and be converted to the highly reactive •OH, or it can be metabolized by myeloperoxidase (MPO) to form hypochlorous acid (HOCl). The reaction of O_2_^●−^ with NO• occurs approximately three times faster than the reaction of O_2_^●−^ with SOD [18,19] and results in reduced NO availability for its biological functions. In mammalian cells, potential enzymatic sources of ROS include NADH/NADPH oxidases and xanthine oxidase, and enzymes involved in the “uncoupling” of eNOS and mitochondrial respiration, as well as other sources. Mitochondrial damage is the common cause of increased ROS from mitochondria. Similarly, if internal antioxidant enzymes (SOD, catalase, and GPX) have decreased in their levels or activities, ROS in the cell will increase [20,21].

**Figure 1:**
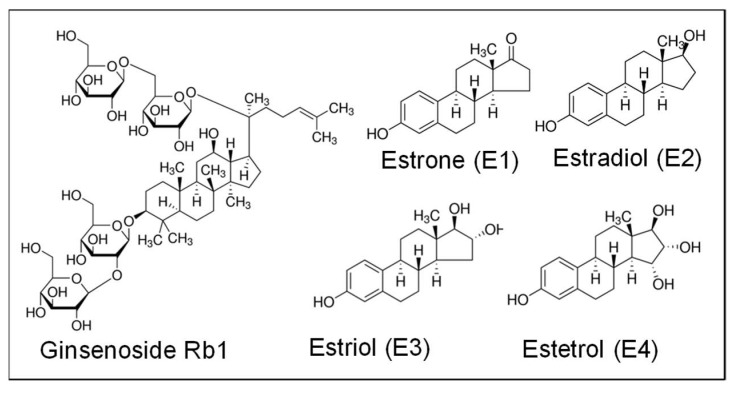
Chemical structures of ginsenoside Rb1 as well as natural forms of human estrogens. Ginsenoside Rb1 possesses 4 trans-ring rigid steroid skeletons with a modified side-chain at C20, C24-C25 double bound, and 4 glucose moieties. Estrone (E1), estradiol (E2), and estriol (E3) are the major naturally occurring forms of estrogens in females. Estetrol (E4) is produced only during pregnancy.

Ginsenosides are the major pharmacologically active ingredients of ginseng [22,23]. More than 40 different ginsenosides have been identified and isolated from the root of *P. ginseng*. Major ginsenosides are Rb1, Rb2, Rc, Rd, Rg3, Rh2, Re, Rf, Rg1, Rg2, and Rh1. Ginsenosides display strong antioxidant effects in animal models and cell cultures [22,23,24]. For instance, ginsenoside Rb1 effectively reduces ROS production and blocks endothelial dysfunction induced by several risk factors including homocysteine, the adipokine resistin, and TNF-α [25,26,27,28]. Ginsenoside Rb1 exerts its antioxidant activity by two major mechanisms: chemical scavenging of certain types of ROS and a receptor-mediated genomic effect on antioxidant protein expression, such as SOD. However, it is not clear how Rb1 exerts its antioxidant function in human endothelial cells. Unlike many other antioxidant mechanisms, the antioxidant mechanisms of ginsenosides may involve both direct ROS scavenging effects and ligand-receptor signaling. Ginsenosides possess 4 trans-ring rigid steroid skeletons with a modified side-chain at C20, which is absent in 17β-estradiol (E2) (Figure 1) [22]. Several ginsenosides have been identified as phytoestrogens; they are able to bind to estrogen receptors [29,30,31,32], glucocorticoid receptors [29,33] and androgen receptors [34].

It is reported that estrogen acts as an antioxidant via stimulation of antioxidant enzymes, thus reducing the production of ROS [35,36,37]. Estrogen also exerts radical scavenging effects by increasing NO production [38], decreasing AT1 receptor expression [39], and inhibiting NADPH oxidase activation [40]. The effects of estrogens are mediated through two receptors, estrogen receptor β (ER-β) and α (ER-α) [41]. Both ERs are widely expressed in different tissue types: ER-α is found in endometrium, breast cancer cells, and epithelium cells; and ER-β has been found in endothelial cells [42]. It has been shown that cultured myometrial and fibroid smooth muscle cells constitutively express ER-α, but not ER-β, while myometrial and fibroid microvascular endothelial cells constitutively express ER-β [43]. ER-α and ER-β bind estrogen with similar high affinity, which results in receptor dimerization [44]. These ligand-activated ERs can mediate the transcription of a range of estrogen target genes by binding to specific DNA sites in gene promoter regions known as estrogen response elements (ERE) or by interacting with other DNA binding proteins, such as the AP1 element, and subsequently modulating gene transcription [41,44]. The major differences between ER-α and ER-β are their tissue distribution, the phenotype of the corresponding knock-out mice, and their transcriptional activities. ER-α and ER-β can mediate opposite transcriptional activities depending on the type of response element in target gene promoters and on other cell-specific factors [45].

In particular, activation of ERs might induce a genomic effect, in which the expression or activity of intracellular antioxidant enzymes could increase to control oxidative stress [46]. We have shown that both ginsenoside Rb1 and estrogen inhibit homocysteine-induced oxidative stress and endothelial dysfunction in porcine coronary arteries [25,47]. Our recent study indicates that Rb1 is a strong scavenger of •OH radical and HOCl [48]. However, Rb1 does not scavenge ONOO^−^, O_2_^●−^ and H_2_O_2_ in cell free systems [48]. In this study, we intend to determine whether Rb1 can interact with ERs and induce the expression of intracellular antioxidant enzyme SOD in endothelial cells to block the endothelial dysfunction caused by HAART drugs. This study will advance our understanding of how ginsenoside Rb1 or its derivatives can potentially help treat or prevent HAART-associated cardiovascular complications in HIV-infected patients and, therefore, may have a significant impact on local and global health.

## 2. Results

### 2.1. Human Endothelial Cells Express ER-β

**Figure 2:**
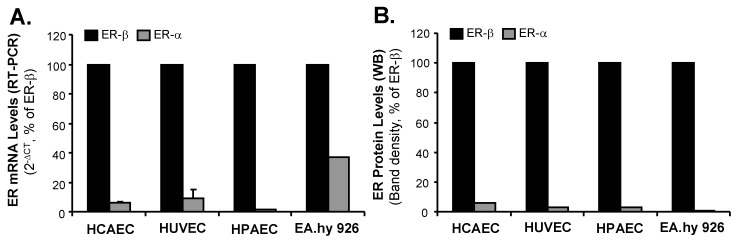
Expression of estrogen receptors (ERs) in human endothelial cells. (**A**) mRNA levels of ER-α and ER-β (Real time PCR). Relative mRNA levels of ER-α and ER-β were calculated as a value of the cycle threshold (*C*_t_), which was normalized to GAPDH mRNA levels (*C*_t_ values) by calculating the 2^[*C*t(GAPDH) − *C*t(ER)]^ value, simply termed 2^−∆*C*t^. For each cell type, mRNA level of ER-β was standardized as 100% (internal control), while the mRNA level of ER-α was compared with that of ER-β; (**B**) Protein levels of ER-α and ER-β (Western blot). A protein (30 μg) sample from each cell line was loaded in the each well of SDS-PAGE (10% polyacrylamide) for electrophoresis. ER-β (59 kDa) and ER-α (65 kDa) protein bands were detected by their specific antibodies. The same blot with the same protein loading was used for detecting both ER-β and ER-α proteins. The density of Western blot bands was analyzed by using NIH ImageJ software. Thus, a positive ER-β band could serve as an internal control for ER-α, which had no band. For each cell type, protein level of ER-β was standardized as 100% (internal control), while the protein level of ER-α was compared with that of ER-β. HCAEC (human coronary artery endothelial cells); HUVEC (human umbilical vein endothelial cells); HPAEC (human pulmonary artery endothelial cells); EA.hy 926 (Immortalized HUVEC cell line); PCR (polymerase chain reaction). WB (Western blot). Human endothelial cells express mainly ER-β and no or very small amounts of ER-α.

Estrogen receptors are present in two forms, ER-β and ER-α. We have observed different ER mRNA levels and different ER proteins among several types of human endothelial cells including human coronary artery endothelial cells (HCAECs), human pulmonary artery endothelial cells (HPAECs), human umbilical vein endothelial cells (HUVECs), and an immortalized HUVEC cell line (EA.hy 926) (Figure 2A). From real time PCR analysis, each cell line had a different cycle threshold (Ct) value of GAPDH compared with other cell lines: HCAEC (17.13), HUVEC (15.05), HPAEC (13.55) and EAhy926 (15.75). These different *C*_t_ values of GAPDH were used separately for calculating 2^−∆*C*t^ values, which represent mRNA levels of ER-α or ER-β in different endothelial cell lines. These endothelial cells mainly express ER-β, but not ER-α. Among these cells, HCAEC and HPAEC had relatively high ER-β expression, while HUVEC and EA.hy 926 had relatively low ER-β expression. To further confirm the expression of ER-β and ER-α, we performed Western blot analysis to detect protein levels of ER-β and ER-α. A protein (30 μg) sample from each cell line was loaded in the each well of SDS-PAGE (10% polyacrylamide) for electrophoresis. ER-β (59 kDa) and ER-α (65 kDa) protein bands were detected by their specific antibodies. The same blot with the same protein loading was used for detecting both ER-β and ER-α proteins. The density of Western blot bands was analyzed by using NIH ImageJ software. Thus, a positive ER-β band could serve as an internal control for ER-α, which had no band. Thus, without GAPDH or β-actin as a loading control, our Western blot densitometry data still demonstrate that expression of ER-β is much higher than that of ER-α in human endothelial cell lines (Figure 2B). All endothelial cells express very limited or no ER-α protein.

### 2.2. Rb1 Blocks Ritonavir-Induced Oxidative Stress through ER-β in Human Endothelial Cells

**Figure 3:**
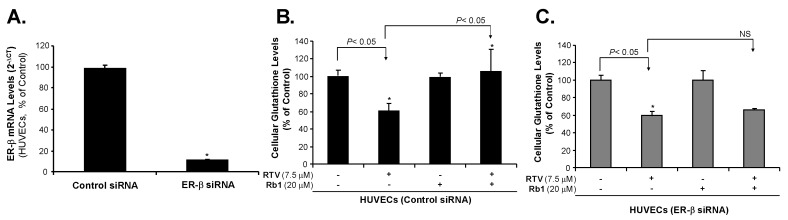
Effect of ER-β silencing, ritonavir (RTV), and ginsenoside Rb1 on ROS levels in HUVECs. (**A**) Effect of ER-β silencing on ER-β mRNA (real time PCR). ER-β silencing was achieved with specific oligonucleatide siRNA as compared with a scramble siRNA as a negative control (100%); (**B**) Cellular glutathione (GSH) levels in HUVECs treated with control siRNA. The results of the cellular GSH assay are inversely proportional to reactive oxygen species (ROS) levels. Cellular glutathione levels were standardized with untreated control cells as 100%. RTV treatment decreased cellular GSH levels, indicating oxidative stress. Ginsenoside Rb1 blocked the effect of RTV in HUVECs treated with control siRNA; (**C**) Cellular glutathione (GSH) levels in HUVECs treated with ER-β siRNA. RTV treatment decreased cellular GSH levels, while ginsenoside Rb1 did not block the effect of RTV in HUVECs in which ER-β had been silenced. Student’s *t*-test was used to compare the control with the treated cells or between two groups. * *p* < 0.05. *n* = 3/group. siRNA (small interfering RNA).

To confirm the role of ER-β in endothelial cells, we silenced ER expression in endothelial cells (Figure 3A). In HUVEC cells, we used a scramble siRNA as a control and ER-β siRNA oligonucleotide to silence ER-β mRNA. We found that ER-β siRNA can effectively silence ER-β expression. Compared with the control, treatment with ER-β siRNA oligonucleotide resulted in a 90% reduction of the ER-β mRNA level.

It is well known that intracellular glutathione (GSH), a three-amino-acid peptide (L-γ-glutamyl-L-cysteinyl-glycine), is an antioxidant found in eukaryotic cells [49,50,51]. Its level has been used as a reliable indicator of cellular oxidative stress. Decreased cellular GSH levels indicate that cells may be under oxidative stress because increased cellular ROS can reduce GSH levels by directly oxidizing its thiol group of cysteine residue [52]. Thus, measuring cellular GSH (reduced form) levels is an effective method to understand cellular ROS levels [53]. The GSH-Glo assay is a luminescent-based assay for the detection and quantification of total content of cellular GSH (reduced form). The assay is based on the conversion of a luciferin derivative into luciferin in the presence of GSH by a glutathione S-transferase (GST) enzyme supplied in the assay kit. Newly formed luciferin is reacted with Ultra-Glo Recombinant Luciferase that generates a glow type luminescence, which is proportional to the amount of cellular GST (reduced form). As a note, cellular GSH levels may be affected by other mechanisms such as altered expression of glutathione-synthesizing or reducing enzymes (Nrf2/ARE pathway) [54]. In the current study, overnight treatment of HUVECs with RTV (7.5 μM) led to a 38% (*p* < 0.05) reduction in GSH levels, indicating that the RTV treatment significantly increased ROS production (Figure 3B). Rb1 alone had no effect on GSH level. Pretreatment for one hour with 20 μM ginsenoside Rb1 before RTV administration increased the level of GSH by 72% over the level measured in response to RTV treatment alone (*p* < 0.05%). Rb1 reversed the GSH level of HUVECs, indicating that Rb1 blocked the formation of ROS. When ER-β mRNA was silenced by ER-β siRNA in HUVECs, treatment with RTV decreased the GSH level by 44%. However, Rb1 did not significantly reverse the RTV effect in HUVECs after silencing ER-β (Figure 3C), indicating that ginsenoside Rb1 requires ER-β for its antioxidant activity in human endothelial cells. These new data are consistent with our previous publications in which Rb1 reduced ROS, including superoxide anion (O_2_^●−^) induced by HAART drugs including RTV demonstrated by ROS specific assays including dihydroethidium (DHE) fluorescence staining and lucigenin-enhanced chemiluminescence assay [11,55,56,57,58,59,60,61].

### 2.3. Rb1 Blocks RTV-Induced Downregulation of SOD1, SOD2 and eNOS through ER-β in Human Endothelial Cells

**Figure 4:**
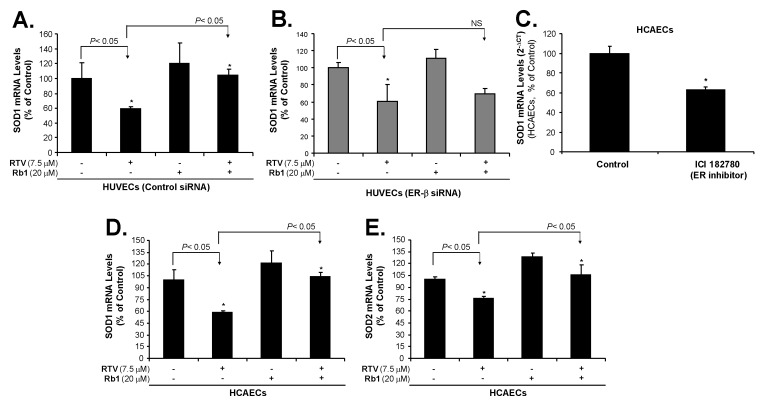
Effect of ER-β silencing, ritonavir (RTV), and ginsenoside Rb1 on the mRNA levels of SOD1 and SOD2 (real time PCR). (**A**) RTV treatment decreased SOD1 mRNA levels, and ginsenoside Rb1 effectively blocked this effect of RTV in HUVECs with control siRNA; (**B**) RTV treatment decreased SOD1 mRNA levels, while Rb1 did not block RTV-induced reduction of SOD1 mRNA levels in HUVECs in which ER-β had been silenced; (**C**) HCAECs were treated with ER inhibitor ICI 182780 (10^−6^ M) or DMSO (negative control) for 24 h, SOD1 mRNA levels were decreased in the ICI-treated group compared with control group, showing a critical role of ER on the maintenance of SOD1 levels; (**D**) RTV treatment decreased SOD1 mRNA levels, and ginsenoside Rb1 effectively blocked this effect of RTV in HCAECs; (**E**) RTV treatment decreased SOD2 mRNA levels, and ginsenoside Rb1 effectively blocked this effect of RTV in HCAECs. Student’s *t*-test was used to compare the control with the treated groups or between two groups. *n* = 3, * *p* < 0.05.

Compared with the control, HUVECs treated with RTV showed a significant reduction in SOD1 mRNA levels (Figure 4A). However, when HUVECs were treated with RTV and Rb1 together, this RTV effect was effectively blocked. In HUVECs treated with Rb1 alone (without RTV), SOD1 mRNA was upregulated, when compared with the control; this indicates that Rb1 can promote SOD1 expression. However, treatment of HUVECs with Rb1 when ER-β was silenced did not dramatically upregulate SOD1 mRNA levels when compared with the cells whose ER-β expression was not silenced (Figure 4B). These data indicate that Rb1 mediates its antioxidant activity via ER-β. To confirm that ER is directly involved in the SOD1 expression, we used ER inhibitor ICI 182780 to treat HCAECs for 24 h, which showed that ICI 182789 significantly reduced SOD1 mRNA levels as compared with negative controls (*p* < 0.05, Figure 4C). Furthermore, RTV treatment decreased SOD1 and SOD2 mRNA levels, and ginsenoside Rb1 effectively blocked this effect of RTV in HCAECs (Figure 4D,E).

**Figure 5:**
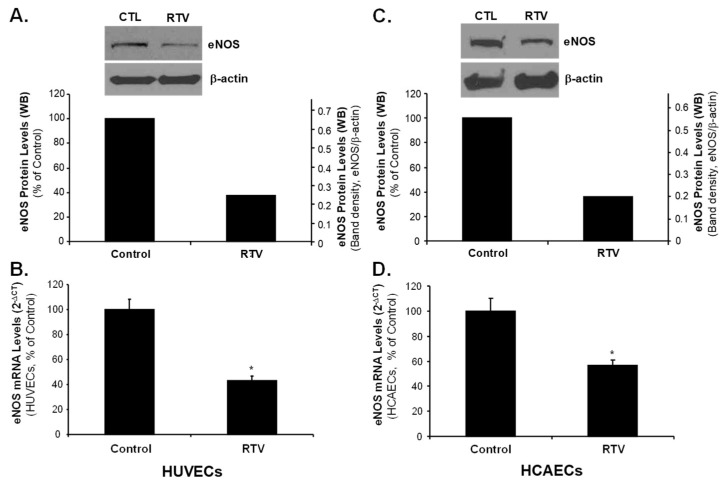
Effect of ritonavir on eNOS expression in human endothelial cells. HUVECs (human umbilical vein endothelial cells) and HCAECs (human coronary artery endothelial cells) were treated with ritonavir (RTV, 15 μM) or DMSO (negative control) for 24 h. The expression of eNOS was determined by Western blot and real-time PCR analysis. (**A**) Protein levels of eNOS (Western blot) in HUVECs. The density of Western blot bands was analyzed by using NIH ImageJ software. Protein level of eNOS in the control cells was standardized as 100%, while protein level of eNOS in RTV-treated cells was compared with that of the control; (**B**) mRNA levels of eNOS (real time PCR) in HUVECs. eNOS mRNA levels were standardized with untreated control cells as 100%; (**C**) Protein levels of eNOS (Western blot) in HCAECs; (**D**) mRNA levels of eNOS (real time PCR) in HCAECs. Student’s *t*-test was used to compare the control with the treated groups. *n* = 3. * *p* < 0.05.

**Figure 6:**
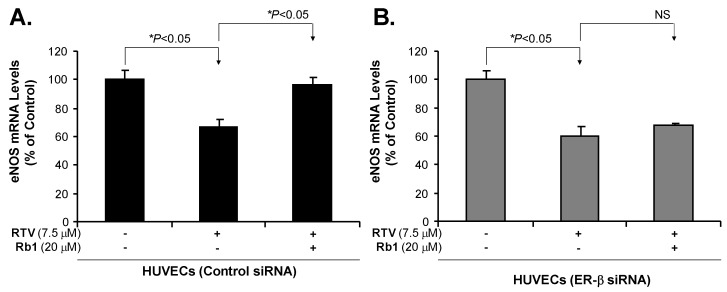
Effect of ER-β silencing, ritonavir (RTV), and ginsenoside Rb1 on the mRNA levels of eNOS in HUVECs (real time PCR). (**A**) eNOS mRNA levels in HUVECs treated with control siRNA. RTV treatment decreased eNOS mRNA levels, and ginsenoside Rb1 effectively blocked this effect of RTV in HUVECs with control siRNA; (**B**) eNOS mRNA levels in HUVECs treated with ER-β siRNA. RTV treatment decreased eNOS mRNA levels, while Rb1 did not block RTV-induced reduction of eNOS mRNA levels in HUVECs with ER-β silencing. Student’s *t*-test was used to compare the control with the treated groups or between two groups. * *p* < 0.05. *n* = 3/group. eNOS (endothelial nitric oxide synthase); HUVECs (human umbilical vein endothelial cells); siRNA (small interfering RNA); PCR (polymerase chain reaction).

Similarly, the treatment with RTV significantly reduced eNOS protein and mRNA levels in both HUVECs and HCAECs as compared with negative controls (*p* < 0.05, Figure 5). Furthermore, HUVECs treated with RTV showed a significant reduction in eNOS mRNA levels, and co-treatment with 20 μM Rb1 restored eNOS expression (Figure 6). However, the effect of RTV was not blocked by Rb1 in HUVECs treated with ER-β siRNA, indicating that ER-β is involved in the gene regulation by Rb1.

### 2.4. Rb1 Binds to ER-β in the Cell Free System

**Figure 7:**
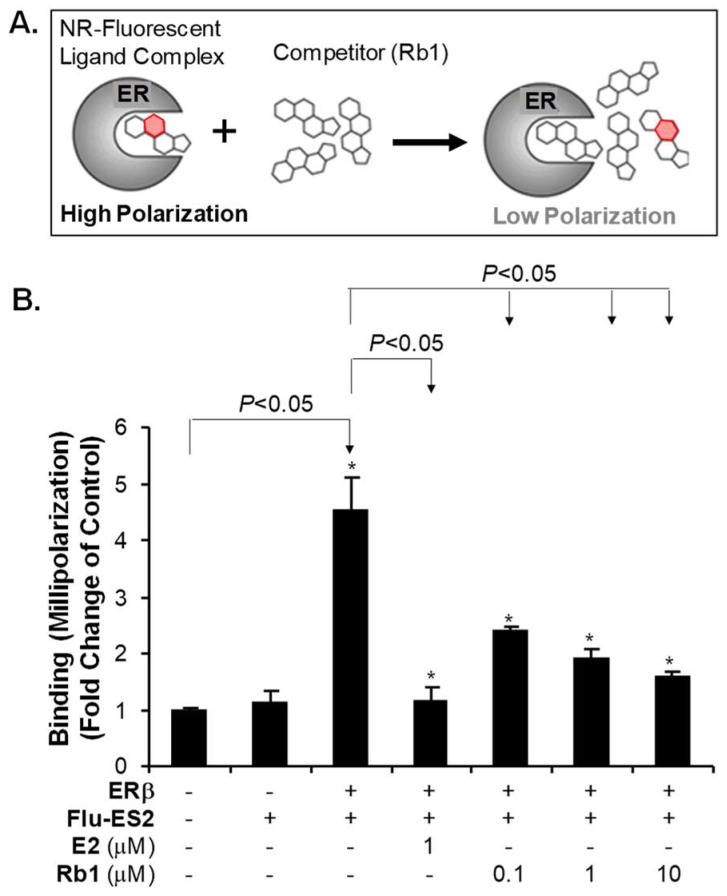
Estrogen receptor (ER) competitor assay. (**A**) Principle of the assay (Invitrogen kit); (**B**) Ginsenoside Rb1 binding assay. E2 was used as a positive control. ER-β binding data were standardized with the untreated control group as 1. Student’s *t*-test was used to compare the control with the treated groups or between two groups. * *p* < 0.05. *n* = 3/group. Three concentrations of Rb1 showed effective binding capability to ER-β. E2 (estradiol).

We showed that RTV treatment caused oxidative stress, which apparently decreased the levels of GSH, SOD mRNA and eNOS mRNA in HUVECs. We further showed that Rb1 can effectively block RTV-induced endothelial dysfunction. However, Rb1 cannot block RTV-mediated dysfunction in HUVECs in which ER-β has been silenced. In order to determine whether Rb1 can bind to ER-β, we performed an ER competition assay. ER and the fluorescent estrogen ligand Fluormone™ ES2 form an ER/Fluormone™ ES2 complex, which shows a high polarization value during its fluorescence lifetime. If Rb1 displaces the Fluormone™ ES2 ligand from ER, the result will show a low polarization value (Figure 7A). We used the change in polarization value in the presence of Rb1 to determine the relative affinity of Rb1 for ER. Control ER-β or Flu-ES2 alone showed low polarization. We found that when ER-β was added to Flu-ES2, without a competitor, the complex showed high polarization, as expected. When we added positive competitor control 17b-estradiol (E2), we observed low polarization, and when we added Rb1 to the ER-β/Fluormone™ ES2 complex, we observed that the polarization value decreased in a concentration-dependence manner, indicating that Rb1 can effectively bind to ER-β (Figure 7B).

### 2.5. Rb1 Activates Transcriptional Activity of ER-β in 293T Cells

**Figure 8:**
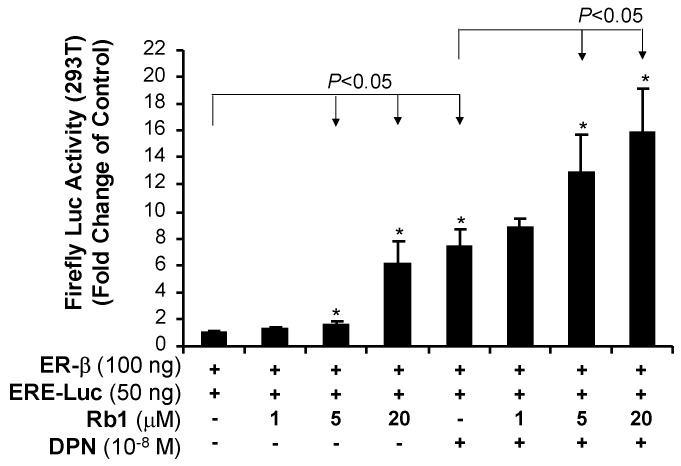
Direct effect of ginsenoside Rb1 and diarylpropionitrile (DPN) on ER-β activation in 293T cells. ER-β cDNA plasmid and ERE-Firefly luciferase reporter plasmid as well as Renilla luciferase reporter plasmid were co-transfected into 293T cells. ERE-Firefly Luciferase activities were normalized with internal control of Renilla luciferase activities. Ginsenoside Rb1 or/and DPN activated ER-β and reporter gene expression in 293T cells, indicating that both Rb1 and DPN can directly activate ER-β for a genomic response. ER-β activation data were standardized with the untreated control group as 1. Student’s *t*-test was used to compare the control with the treated group or between two groups. * *p* < 0.05. *n* = 3/group. ER-β (estrogen receptor β); DPN (specific ER-β activator); ERE (estrogen responsive element); 293T cells (cell line derived from human embryonic kidney cells).

To further confirm whether Rb1 directly activates ER-β, we co-transfected an ER-β cDNA plasmid and a reporter gene plasmid containing estrogen response element (ERE) and Firefly luciferase cDNA as well as Renilla luciferase reporter plasmid (internal control) into 293T cells. We found that, relative to the control cells, Rb1 activated ER-β for reporter gene expression in 293T cells (Figure 8). Diarylpropionitrile (DPN), a specific activator of ER-β, which we used as a positive control, activated ER-β activity significantly. When Rb1 and DPN were used together, the result showed an additive effect on the activation of ER-β, indicating that both Rb1 and DPN can directly activate ER-β and produce a genomic response.

## 3. Discussion

In the current study, we found that all four types of human endothelial cells tested had a differential expression of mainly ER-β, at both mRNA and protein levels, and that the level of ER-α mRNA was very low and ER-α proteins were not detectable. RTV treatment significantly increased ROS production and decreased the expression of SOD1/2 and eNOS in HUVECs and HCAECs, while Rb1 effectively blocked these effects of RTV. When ER-β was silenced in HUVECs, the effects of Rb1 on blocking RTV-induced ROS production and downregulating eNOS were inhibited. Rb1-induced upregulation of SOD1 was also inhibited in ER-β-silenced HUVECs. Rb1 specifically bound to and activated ER-β transactivation activity in 293T cells.

It has been shown that oxidative stress plays a pivotal role in endothelial dysfunction, cardiovascular diseases, and other pathogenic conditions [62,63,64]. It has also been reported that free radicals can play a key role in atherosclerotic plague formation and endothelial dysfunction [62,65], and that RTV can cause endothelial dysfunction in human endothelial cells, and porcine coronary, porcine pulmonary, and carotid arteries [55]. In this study, we observed a significant decrease of GSH level in RTV-treated HUVECs. A change in GSH levels is important for assessing ROS levels. ROS can cause a drop in GSH levels either by oxidizing the thiol group of cysteine residue. GSH is a major antioxidant, which can interact with several ROS including O_2_^●−^ [66], H_2_O_2_ and HOCl [67]. Intracellular ROS could be interchanged; for example, O_2_^●−^ could become H_2_O_2_ by SOD, while H_2_O_2_ could become HOCl by MPO [16]. Therefore, RTV treatment of HUVECs results in oxidative stress, such as increased production of superoxide anion, by either inhibiting SOD expression or other ROS conversion enzymes such as catalase and glutathione peroxidase, or by activating superoxide-generating enzymes such as xanthine oxidase and NADPH oxidase. In this study, we observed the RTV-induced downregulation of SOD1/2 and eNOS expression. SOD1, one of the three forms of human SOD enzyme located in the cytoplasm, dismutes superoxide anion to oxygen and hydrogen peroxide. Therefore, RTV treatment of HUVECs downregulates the expressions of SOD1 and eNOS enzymes; SOD1 downregulation implies a high level of superoxide, resulting in a significant decrease of GSH levels in RTV-treated HUVECs. SOD2 (mitochondrial SOD), also known as manganese-dependent superoxide dismutase (MnSOD), was also studied in the current study. Treatment of RTV significantly reduced mRNA levels of both SOD1 and SOD2 in HCAECs, while RB1 can effectively block this effect of RTV. The superoxide anion subsequently converts to other ROS or rapidly reacts with another biological species, such as endothelium-derived nitric oxide (NO), making a toxic peroxynitrite. The later reaction results in reduction or loss of endothelium-dependent vasorelaxation and increase of other atherogenic processes.

The effects of estrogens are mediated through two receptors, estrogen receptor (ER-β) and (ER-α) [41]. Both ERs are widely expressed in different tissue types; however, endothelial cells differentially express ER-β [42,43]. In this study, we have observed that several types of human endothelial cells (HCAEC, HUVEC, HPAEC and EA.hy 926) mainly express ER-β in different amounts, but not ER-α. Our results are consistent with previous publications [42,43]. For example, human endothelial cells only express ER-β, but not ER-α [68]; ER-β mediates signal transduction pathways in human endothelial cells [69,70]. Endothelial ER-β plays a role in mediating systemic blood pressure in animal models [71]. Although both ER-β and ER-α bind to 17β-estradiol (E2) with similarly high affinities, they show differences in binding to some other steroidal ligands, have distinct tissue distributions, regulate separate sets of genes, and may oppose each other’s actions in some instances [45]. One important function of estrogens is to reduce oxidative stress, which has been observed in humans, animal models, and cell cultures. This process is thought to occur through several mechanisms, such as upregulation of antioxidant enzyme SOD, thus reducing the production of ROS [35]; by exerting radical scavenging effects through increasing NO production [38]; decreasing AT1 receptor expression [39]; and inhibition of NADPH oxidase activation [40]. Estrogens, however, do not act as chemical antioxidants [72]. Several phytoestrogens, such as ginsenosides, also show estrogen-like antioxidant activities correlating with increase in SOD expression [46,73,74]. However, there are no reports describing a ginsenoside-ER signaling pathway to regulate the SOD promoter activities. Our previous studies in vascular cells clearly showed that Rb1 can effectively reduce ROS, including superoxide anion (O_2_^●−^) induced by several cardiovascular risk factors such as homocysteine [25], adipokine resistin [26,28] and TNF-α as well as HAART drugs [9,10,55]. Our recent study indicates that, in the cell free system, Rb1 chemically scavenges •OH and HOCl, but not O_2_^●−^ [48]. However, in live human endothelial cells, Rb1 was able to reduce O_2_^●−^ levels; this suggests that Rb1 may do so through another pathway, such as ER-mediated genomic signaling, causing the increase of SOD expression. To confirm the role of ER-β on ROS production and SOD expression in endothelial cells, we effectively silenced ER-β mRNA with siRNA oligonucleotide in HUVECs. In this study, RTV treatment of cells significantly increased ROS, (thereby decreased GSH), downregulated expressions of SOD1/2 and eNOS enzymes, we observed that Rb1 significantly inhibit the deleterious effect of RTV. However, Rb1, to reverse the deleterious effect of RTV in HUVECs, was significantly reduced after silencing ER-β (Figure 3, Figure 4, Figure 6), indicating that ginsenoside Rb1 requires ER-β of its antioxidant activity in human endothelial cells. Likewise, treatment of endothelial cells with Rb1 when ER-β was silenced did not as dramatically upregulate SOD1 mRNA levels, when compared with when ER-β expression was normal. These results indicate that ER-β played an important role.

To further confirm that Rb1 can directly activate ER-β, we co-transfected an ER-β cDNA plasmid and a reporter gene plasmid containing estrogen response element (ERE) with Firefly luciferase cDNA plasmid into 293T cells, and observed that, indeed, Rb1 can activate ER-β-dependent reporter gene expression. DPN showed an additive effect with Rb1 on ER-β activation. Furthermore, our ER competition assay indicated that Rb1 can effectively bind to ER-β in a concentration-dependent manner. Our data strongly indicate that Rb1 is able to effectively bind to ER-β and induce a genomic response leading to SOD upregulation via Rb1-ER-β signaling pathway in human endothelial cells. To the best of our knowledge, we, for the first time, have shown a causal relationship between Rb1-ER-β interaction and SOD expression in human endothelial cells.

Ginsenoside Rb1 has a four-ring, steroid-like structure with sugar moieties attached [75]. Like steroids, Rb1 is lipophilic [76] and it enters the nucleus by simple diffusion to control gene transcription by binding to specific intracellular receptors. Several studies have confirmed that Rb1 exerts an estrogen-like effect by binding to estrogen receptors (ERs) [77,78,79]. Indeed, ginsenoside Rb1 can activate ERs in a ligand-independent manner by a variety of stimuli, including the insulin-like growth factor I [80], epidermal growth factor [81] and serum [82]. In human endothelial cells, Rb1 selectively binds to ER-β, but not the ER-α [83,84]. These data are consistent with our results. However, in a human breast cancer cell line MCF-7, Rb1 seems to bind both ER-α and ER-β receptors [85]. In general, ginsenoside Rb1 is considered as a relatively weak phytoestrogen as compared with 17β-estradiol (E2). Effective concentrations of Rb1 reported in cell cultures range from 0.2 to 50 μM, while E2 is effective in a range of 1–100 nM [83,85]. Thus, estrogenic activity of Rb1 is in the micromolar range.

In our study, we have confirmed that ER-β, but not ER-α, is a major receptor expressed in four types of human endothelial cells (Figure 2). The concentration (20 μM) of Rb1 selected to test its functions to block the effect of ritonavir was based on our published data on Rb1 [11,25,56,57,58,86]. For the receptor binding assay and promoter reporter assay, we used lower concentrations of Rb1 (0.1, 1, 5, 10 and 20 μM), showing positive results (Figure 7 and Figure 8).

We have done extensive studies to confirm that the ritonavir-induced endothelial dysfunction is mediated by oxidative stress; accordingly, several natural substances with antioxidant activities effectively block ritonavir-induced oxidative stress in endothelial cells and porcine coronary artery rings including seleno-L-methionine (SeMet), epigallocatechin gallate (EGCG), curcumin, capsaicin, equol, nordihydroguaiaretic acid (NDGA), and dihydroxybenzyl alcohol (DHBA) [55,57,58,59,60,61,87,88,89,90]. In addition, we have also shown that ginsenoside Rb1 effectively blocks the ritonavir-induced oxidative stress and endothelial dysfunction [11,56]. However, little is known about the underlying antioxidant mechanisms of ginsenoside Rb1. In a cell free system, we have found that Rb1 can significantly and selectively reduce hydroxyl radical (–OH) and hypochlorous acid (HOCl), two of the strongest ROS, while it has no or limited scavenging activity against superoxide anion (O_2_^●−^), peroxynitrite (ONOO^−^), and hydrogen peroxide (H_2_O_2_) [48]. In the current study, we hypothesized that ginsenoside Rb1 could reduce the superoxide anion (O_2_^●−^) indirectly by increasing the intracellular antioxidant enzyme SOD through its estrogenic activity. Indeed, we have shown that Rb1 binds to ER-β (Figure 7 and Figure 8), upregulates SOD1 expression (Figure 4), and reduces cellular O_2_^●−^ levels (Figure 3B) in human endothelial cells. Thus, ginsenoside Rb1 has different mechanisms of antioxidants such as direct scavenging of –OH and HOCl and indirect scavenging of O_2_^●−^ by SOD. In the current study, we also encountered some technique difficulties such as detailed protein analysis of ERs, eNOS and SOD1/2, which showed some limitations. Furthermore, we used the GSH-glo kit to measure the reduced form of GSH as an indicator for cellular oxidative stress. With the current results, we believe that ritonavir induces ROS production, which may exhaust intracellular GSH (the reduced form); however, we cannot rule out the possibility that ritonavir may affect the glutathione biosynthesis of GSH, which is an unknown issue.

## 4. Materials and Methods

### 4.1. Chemicals and Reagents

The following reagents were obtained from Sigma-Aldrich (St Louis, MO, USA): diarylpropionitrile (DPN), 17β-estradiol (E2), glutathione, and ER inhibitor ICI 182780. Ginsenoside Rb1 (molecular weight, 1109.29, purity ≥ 98% by HPLC, white powder, soluble in water) was obtained from LKT Laboratories, Inc. (St. Paul, MN, USA). A stock solution of ginsenoside Rb1 (purity ≥ 98%) was prepared in pure water at a concentration of 10 mM. RTV was obtained from AIDS Research and Reference Reagent Program (NIH). EBM-2 medium was obtained from Lanza. Dulbecco’s modified Eagle’s medium (DMEM), the PolarScreen ER-β competitor Assay, and the PolarScreen ER-α competitor Assay kits were obtained from Life Technologies (Grand Island, NY, USA). ER-β siRNA and scrambler control siRNA were obtained from Santa Cruz Biotechnology, Inc. (Santa Cruz, CA, USA). GSH-Glo™ Glutathione Assay kit and Luciferase Reporter Assay Kit were obtained from Promega (Madison, WI, USA).

### 4.2. Cell Culture

Human coronary artery endothelial cells (HCAECs), human pulmonary artery endothelial cells (HPAECs), human umbilical vein endothelial cells (HUVECs), and an immortalized HUVEC cell line (EA.hy 926) were used. All endothelial cells were purchased from Lonza. The cells were used at passage 4 to 5. When monolayers were 80 to 90% confluence in six-well culture plates, they were treated with DMSO (control) or RTV with or without Rb1 at the concentrations described above, for 24 h at 37 °C. In these cell cultures, we then studied the expression of ERs, SOD1, SOD2 and eNOS as well as superoxide anion production. Expression of ERs was determined with real time PCR and Western blot. ER-β silencing was achieved with specific oligonucleotide siRNA.

### 4.3. Real Time PCR

When endothelial cell monolayers growing in 12-well plates were 80% confluent, we collected the cells via trypsin digestion. We then extracted total cellular RNA with an RNAqueous-4PCR kit (Ambion, Austin, TX, USA), and converted the RNAs into cRNA with the iScript cDNA Synthesis Kit (Bio-Rad, Hercules, CA). We detected mRNA levels of ERs, SOD1, SOD2 and eNOS with real-time PCR, and used glyceraldehyde-3-phosphate dehydrogenase (GAPDH) as an internal control to account for variations in mRNA loading. Real-time PCR was performed in an iCycler iQ real-time PCR detection system (Bio-Rad, Hercules, CA, USA). Primers for ERs, SOD1/2 and eNOS were designed via the Beacon Designer 2.1 software (Bio-Rad) as previously reported [9,10,11]. Relative mRNA levels of ERs, SOD1/2, and eNOS were calculated as a value of cycle threshold (Ct), which was normalized to GAPDH mRNA levels for all endothelial cells and presented as 2^[Ct(GAPDH) − Ct(specific gene)]^, simply termed 2^−∆Ct^ as previously described [9,10,11]. The primer sequences for the ER gene were: HESR-2-F: AGAGTCCCTGGTGTGAAGCAAG; HESR-2-R: GACAGCGCAGAAGTGAGCATC; HESR-1-F: CCACCAACCAGTGCACCATT; HESR-1-R: GGTCTTTTCGTATCCCACCTTTC.

### 4.4. Western Blot

Endothelial cells were harvested when monolayers were 80% confluent and subsequently lysed for 30 min in ice. Cell lysates were then collected after centrifugation at 15,000 rpm for 10 min at 4 °C. Equal amounts of endothelial proteins (15 µg or 30 μg) were first resolved electrophoretically with one-dimensional SDS-PAGE (10% polyacrylamide) and then electrophoretically transferred into nitrocellulose. eNOS and ER proteins were detected with mouse anti-eNOS and anti-ER monoclonal antibodies, respectively, from Santa Cruz Biotechnology, Inc. (Santa Cruz, CA, USA).

### 4.5. Glutathione (GSH) Assay

HUVECs monolayers that were 80% confluent were organized in groups and received the following treatments: no treatment (control), RTV (7.5 μM) only, ginsenoside Rb1 (20 μM) only, and RTV (7.5 μM) after pretreatment for one hour with ginsenoside Rb1 (20 μM). The cells were harvested by scrapping the monolayer in PBS after being washed 2 times with 1x cold PBS. Cellular oxidative stress was indirectly determined by measuring glutathione levels with a GSH assay kit (Promega, Madison, WI), as per the manufacturer’s instructions.

### 4.6. ER-β Luciferase Reporter Assay

In the current study, we used Promega’s Dual-Luciferase^®^ Reporter Assay Systems, which pGL4 ERE-Firefly luciferase reporter plasmid and pGL4 SV40 promoter-driven Renilla luciferase reporter plasmid as a control vector. ER-β plasmid cDNA, ERE-Firefly luciferase reporter plasmid cDNA as well as Renilla luciferase reporter plasmid were co-transfected into 293T cells (cell line derived from human embryonic kidney cells). Cells were harvested in reporter lysis buffer at 24 h post-transfection, and lysates were assayed for luciferase activity with a dual luciferase assay kit (Promega, Madison, WI), as per the manufacturer’s instructions. Luciferase activities were normalized according to the ratio of firefly to Renilla luciferase activities. Because the Renilla luciferase reporter vector lacks ERE, it served as an internal control for ERE-responsible Firefly luciferase reporter vector.

### 4.7. Rb1-ER-β Binding Assay

Rb1-ER-binding was determined with the estrogen receptor competitor assay kit (Life Technologies), as per the manufacturer’s instructions. Briefly, ER was added to a fluorescent estrogen ligand to form an ER/Fluormone™ ES2 complex. This complex was then added to individual test compounds, such as ginsenoside Rb1, in multi-well plates. The polarization of fluorescence of the complex was then measured with the Ultimate Microplate Reader (POLARstar Omega). The change in the polarization value in the presence of test compounds was used to determine the relative affinity of that test compound for ER. We used 17β-estradiol (E2, 1 μM) as a positive control.

### 4.8. Statistical Analysis

All data are presented as the mean ± SEM. Student’s *t*-test was used to make comparisons between two groups. A *p* value < 0.05 was regarded as significant. All statistical analyses were performed by using Microsoft Excel 2016.

## 5. Conclusions

**Figure 9:**
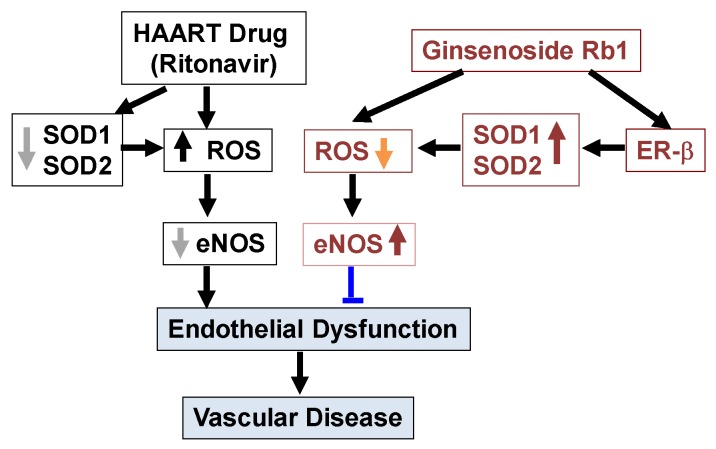
Conclusion of the current study. The current study demonstrates that ritonavir (RTV), an HAART drug, can cause oxidative stress and endothelial dysfunction by increase of ROS production and decrease of expression of SOD1, SOD2 and eNOS in human endothelial cells. Thus, RTV-induced oxidative stress and endothelial dysfunction could contribute to cardiovascular disease. On the other hand, ginsenoside Rb1 can effectively block the effects of RTV via a unique mechanism by which Rb1 specifically binds to and activates ER-β and induces upregulation of SOD1, SOD2 and eNOS in human endothelial cells. Rb1 can also directly scavenge ROS.

Ginsenoside Rb1 can effectively block RTV-induced oxidative stress and downregulation of SOD and eNOS via a unique mechanism by which Rb1 specifically binds to, activates ER-β and induces SOD upregulation in human endothelial cells (Figure 9). Thus, the antioxidant activity of ginsenoside Rb1 may be mediated by intracellular antioxidant SOD1/2. This study significantly advances our understanding of the molecular mechanisms of ginsenoside Rb1 and suggests that Rb1 may have clinical applications in the treatment of cardiovascular disease.
